# OPV strains circulation in HIV infected infants after National Immunisation Days in Bangui, Central African Republic

**DOI:** 10.1186/1756-0500-3-136

**Published:** 2010-05-18

**Authors:** Alexandre Manirakiza, Emmanuella Picard, Richard Ngbale, Didier Menard, Ionela Gouandjika-Vasilache

**Affiliations:** 1 Virology Unit, Institut Pasteur de Bangui, Avenue Pasteur, BP 923, Bangui, Central African Republic; 2 Foyer de Charité, Bangui, Central African Republic; 3 Malaria Unit, Institut Pasteur de Madagascar, Antananarivo, Madagascar

## Abstract

**Background:**

Humans are the only host of polioviruses, thus the prospects of global polio eradication look reasonable. However, individuals with immunodeficiencies were shown to excrete vaccine derived poliovirus for long periods of time which led to reluctance to prolong the vaccination campaign for fear of this end result. Therefore, we aimed to assess the duration of excretion of poliovirus after the 2001 National Immunization Days according to Human immunodeficiency virus status.

**Findings:**

Fifty three children were enrolled. Sequential stool samples were collected in between National Immunisation Days rounds and then every month during one year. Children were classified into 2 groups: no immunodepression (n = 38), immunodepression (n = 15) according to CD4+ lymphocytes cells count. Thirteen poliovirus strains were isolated from 11 children: 5 Human immunodeficiency virus positive and 6 Human immunodeficiency virus negative. None of the children excreted poliovirus for more than 4 weeks. The restriction fragment length polymorphism analysis showed that all strains were of Sabin origin including a unique Polio Sabine Vaccine types 2 and 3 (S2/S3) recombinant.

**Conclusions:**

From these findings we assume that Human immunodeficiency virus positive children are not a high risk population for long term poliovirus excretion. More powerful studies are needed to confirm our findings.

## Findings

### Introduction

Humans are the only host of polioviruses, thus the prospects of global polio eradication look reasonable [1,2]. However the discovery, after years of massive use of oral polio vaccine (OPV), of individuals with immunodeficiencies who were shown to excrete vaccine derived poliovirus (VDPV) for long periods of time led to a reluctance to prolong the vaccination campaign for fear of this end result [3]. Considering the immunodeficiency that prevails in Human immunodeficiency virus (HIV) patients, long term poliovirus excretion would be likely [4,5]. In Africa the OPV is used in mass vaccination campaigns during National Immunization Days (NIDs) notwithstanding HIV status of children. Two recent studies showed that HIV-infected children have low persistence of antibodies to vaccines used in the Expended Program on Immunization (EPI) including OPV [6,7].

In Central African Republic (CAR), it have been assessed that the prevalence of HIV infection is 6% in general population [8] thus it would be of interest to study the impact of HIV infection on poliovirus excretion in infants of 0 to 5 years of age receiving OPV during NIDs in Bangui. Very few studies have been conducted and no persistent excretion of poliovirus has been reported among HIV infected people [9,10] except two vaccine derived polioviruses isolated from 2 HIV-infected children in South Africa [4,5]. Therefore we studied the duration of excretion of poliovirus after the 2001 NIDs according to HIV status.

## Materials and methods

### Population

This survey was achieved within "Foyer de Charité" center from October 2001 until June 2002. This health structure is set up by the Catholic Church and take health care of the children descended of the very poor families in return for a weak financial involvement (2 US$ per child). All children included in this study were under 5 years old. The informed consent of parents or legal tutors was obtained before inclusion in the study. One blood sample (for confirmation of HIV status and CD4+ count) and stool samples were collected before NIDs. All enrolled infants received 3 doses of OPV during the 3 rounds of NIDs. Sequential stool samples were collected in between NIDs rounds and then every month for one year period from the enrolled children. HIV+ positives infants were identified and followed up for opportunistic diseases at this health care structure. Children who did not present at the scheduled day were followed up at home when possible. All samples were processed at Institut Pasteur de Bangui. The date of the last OPV dose administrated was noted.

### Status of human immunodeficiency virus-infected children

According to their HIV status and CD4+ count, the children were classified into 2 groups: i) group A no immunodepression (n = 38; 5 HIV+ and 11 HIV-); ii) group I immunodepression (n = 15; 11 HIV+ and 4 HIV-). Children less than 12 months of age were considered as immunodepressed if the CD4+ count was <500/mm3, and children of more than 12 months of age were considered immunodepressed if the CD4+ was <750/mm^3 ^[11]. Western blot tests (new Lav blot I^©^, BioRad, Marne la Coquette, France) were carried out at Institut Pasteur de Bangui to confirm the HIV status of children. Tests were performed using the same sample to determine the CD4 count. Haematological analysis and CD4 T-cell counts were carried out with a Coulter AcT Diff 2 Analyser and a FACSCalibur Flow Cytometer (Becton Dickinson Immunocytometry Systems, San Jose, CA, USA), as previously described [12].

### Virus isolation and identification

Viruses were isolated and identified according to World Health Organization (WHO) Polio Laboratory Network Standard Protocols [13]. Internal quality-control procedures associated with these methods were implemented. Briefly, stool extracts were inoculated on the following cell lines: RD (human rhabdomyosarcoma derived cells), Hep2 (human epidermoid cancer cells) and murine L20B (a transgenic mouse L cells). The latter cell line was used to distinguish polioviruses from non-polio enteroviruses [14]. Positive RD and Hep2 cell cultures were passaged in L20B to separate poliovirus and non polio enteroviruses. Isolates of poliovirus were identified by neutralization tests with standardized pools of hyperimmune equine serum. Tissue culture infectious dose can be determined, using the double or triple cell-line system. The cells were discarded every 15 passages, as recommended by WHO, in order to ensure high cell sensitivity to enteroviruses and especially for poliovirus. Sensitivity tests were conducted at passage 7 by titration of reference Sabine strains. If the titre is within ±0.5log10 of the expected reference value, it is considered that there is no decline in cell-line sensitivity.

### Reverse Transcriptase- Polymerase chain reaction (RT-PCR) and multiple restriction fragment length polymorphism (RFLP) analysis

Viral ribonucleic acid (RNA) was extracted using the Quiaquick kit (QIAGEN^®^). Three different genomic regions of the poliovirus genome, namely, VP3-VP1 (nucleotides positions 1913 to 2881 according to Sabin 1 numbering), VP1-2A (nucleotides 2870 to 3648) and 3D-3'UTR (nucleotides 6536 to 7441) were targeted. RT- PCR was followed by a multiple RFLP analysis using four restriction enzymes (DdeI, DpnII, RsaI and HinfI). These regions were amplified using nucleotides primers UG24 and UC1, UG19 and UC13 and UG17 and UC10 respectively [15,16].

### Statistical analysis

Data were managed using EpiInfo Software 3.3.2 version (Centres for Disease Control and Prevention, USA). Means of virus isolated were compared according to the immune status of the patient using Mann-Whitney U test.

### Nucleotides sequencing analysis

Nucleotides sequencing of VP1-2A region PCR products was performed with the same primers used for RT-PCR and with internal primers UG1 and UC11. PCR products were sequenced following purification with a Qiaquick spin column purification kit (QIAGEN) immediately after amplification. Sequencing reactions were performed with a BigDye Terminator cycle sequencing ready kit (version 1.1) according to the recommendations of Applied Biosystems. Nucleotide sequences were aligned and compared using Clustal W program [17].

## Results

A total of 117 children were eligible before the first round of the NIDs. Out of 117 children, 53 (45.3%) received the all course of 3 OPV doses during of the NIDs (mean age, 56.3 months; male/female ratio, 0.8) and were enrolled in the study.

**Figure 1:**
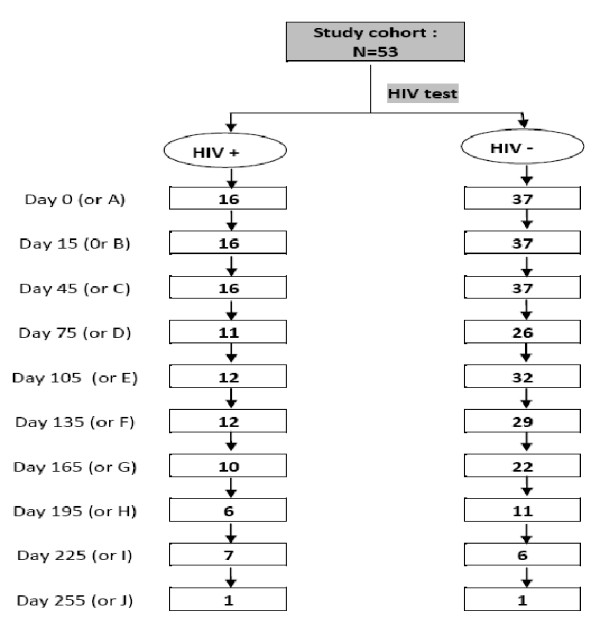
**Sequential stools collection flow chart from the cohort study from Day 0 of follow up**.

HIV test was positive among 16 out of 53 (30.2%) children and negative results were observed in 37 out of 53 (69.8%) children. From the 53 children who have received 3 doses of OPV, a total of 345 stools samples were collected during the follow-up. The total number of stool samples expected for each case was 10. But the number of stools samples we collected significantly decreased during the follow up because of no compliance to indicated visits schedules and lost of follow up at home (home changes to addresses not easily accessible). The mean of the stool sample collected from each case was of 7 and 6 for the HIV+ and HIV- groups respectively (Figure 1).

A proportion of 36.8% of them were positive for an enterovirus (127 viruses isolated from 345 stools). Six poliovirus were isolated on the 107 stools colleted from HIV+ children (5.6%) while five poliovirus were isolated on the 238 stools colleted from HIV- children (2.1%) (*P *value = 0.185). Non Polio Enteroviruses (NPE) were excreted in a proportion of 31.8% (34/107) from all HIV+ children and 34.4% (82/238) from all HIV- children (*P *value = 0.626) (Table 1).

**Table 1:** Viruses isolated according to sample collection session and HIV status from the study cohort (53 children).

	**Non polio enteroviruses**		**Poliovirus**		**Negative**	
			
	**HIV+**	**HIV-**	**HIV+**	**HIV-**	**HIV+**	**HIV-**
		
A	8	16	0	2	8	19
B	3	14	1	2	12	21
C	2	8	2	0	12	29
D	2	6	3	1	6	19
E	4	12	0	0	8	20
F	6	14	0	0	6	15
G	5	4	0	0	5	18
H	0	3	0	0	6	8
I	4	5	0	0	3	1
J	0	0	0	0	1	1
		
Total	34	82	6	5	67	151

A = sample collection before NID's: Day 0, B= sample collection between the 1^st ^and 2^nd ^round of NID's (Day 15), C= sample collection between the 2^nd ^and 3^rd ^round (Day 45), D, E, F, G, H, I, J = sequential sample collection after the 3^rd ^round at one.

Only two HIV- children excreted poliovirus before the NIDs as they received the routine vaccination during the month preceding the first stool sample collection.

All the 15 children with immunodeficiency (group I) secreted an Enterovirus, and 11 of them secreted a virus more than two times. Thirty four out 38 children with no immunodeficiency (group A) secreted an Enterovirus, and 13 of them secreted a virus more than two times. None of the children excreted poliovirus for more than 4 weeks. Only two HIV- children excreted Poliovirus type 1 and 2 strains two times consecutively. There was no statistically significant difference of excretion of an Enterovirus according to HIV serologic (Fisher exact test value = 0,069) and immune level status (U-test *P *values > 0,05 for both HIV+ and HIV- groups) (Tables 1 and 2).

**Table 2:** Number of virus isolated (polio and NPENT together) according to the immune status of the children at the time of inclusion.

	HIV-/A	HIV+/A	HIV-/I	HIV+/I
A	17	3	1	5
B	13	1	3	3
C	7	1	1	3
D	5	2	2	3
E	10	2	2	2
F	10	2	4	4
G	3	1	1	4
H	3	0	0	0
I	3	1	2	3
J	0	0	0	0

Total	71	13	16	27

Patients have been classified according to the CD4 count: i) group A no immunodepression (n = 37); ii) group I immunodepression (n = 16). Children under 12 month of age were considered as immunodepressed if the CD4+ count was <500/mm3, and children of more than 12 month of age were considered immunodepressed if the CD4+ was <750/mm3 [11].

RFLP analysis showed that all strains were of Sabin origin, including a unique S3/S2 recombinant. Sequencing of Poliovirus type 1 and Poliovirus type 3 strains, both isolated from HIV+ infants, showed more than 99% homology with homotypic Sabine strains.

## Discussion

Literature reports that both cellular and humoral immune responses are intact early in life in most children infected with HIV [18,19]. Although HIV infected individuals may shed other enteric viruses for prolonged periods, the lack of persistent poliovirus vaccine excretion is consistent with their ability to develop immunity after vaccination. Even after deterioration of CD4 cell counts, these children retained sufficient immunologic memory to prevent persistent infections from repeated exposures to poliovirus [20,21].

The main aim of our study was to evaluate the risk of prolonged OPV circulation in a population of children receiving massive doses of OPV accordingly to their HIV status. Our findings showed no trend of a prolonged Poliovirus excretion in HIV+ and HIV- children groups.

The relatively small size of the cohort does not allow exclusion of the possibility that a small proportion of HIV infected children may develop prolonged excretion, especially since antiretroviral treatments were introduced in CAR and prolonged the duration of life of these children.

Studies conducted on 28 HIV-infected adults population exposed to vaccinated children in CAR and 419 adults in Cote d'Ivoire following anti-poliovirus vaccination campaigns showed no evidence of prolonged Enterovirus excretion [9,10].

In most industrialized countries, inactivated polio vaccine (IPV) is administrated to individuals with immunodeficiency disorders because the potential risk of vaccine associated paralytic poliomyelitis (VAPP). Nevertheless, the limited data available indicate a risk of VAPP in children with HIV and OPV which is generally administrated to all children regardless their HIV status in low income countries [4,5]. In the past 40 years only 23 persons with IgG deficiency disorders have been found with prolonged poliovirus excretion. Active search for additional persons with prolonged poliovirus excretion among persons with known IgG deficiency disorders revealed no new cases [3,22].

## Conclusion

The small size of the cohort do not allow us to draw a definitive conclusion, but indicates that excretion of poliovirus among HIV infected children is present and such short term excretion is unlikely to be a source of reintroduction of neurovirulent poliovirus following the cessation of OPV use in Central African Republic. More powerful studies are needed to confirm our findings.

## Competing interests

The authors declare that they have no competing interests.

## Authors' contributions

IGV conceived the study, did the data management and paper draft writing with substantial contributions of DM. Field study monitoring, data analysis and interpretation was achieved by AM. The medical and nutritional care of the children was achieved by EP and RN. All authors read and approved the final manuscript.

## References

[B1] Alexander JP Jr Gary HE Jr Pallansch MA Duration of poliovirus excretion and its implications for accute flaccid paralysis surveillance: a review of the literature J Infect Dis 1997 175 S176 S182 10.1093/infdis/175.supplement_1.s176 9203713

[B2] Dowdle WR Birmingham ME The biologic principles of poliovirus eradication J Infect Dise 1997 175 S286 S292 10.1093/infdis/175.Supplement_1.S286 PMC7110371 9203732

[B3] Kew OM Sutter RW de Gourville EM Dowdle WR Pallansch MA Vaccine-derived polioviruses and the endgame strategy for global polio eradication Annu Rev Microbiol 2005 59 587 635 10.1146/annurev.micro.58.030603.123625 16153180

[B4] Pavlov DN Van Zyl WB Kruger M Blignaut L Grabow WO Ehlers MM Poliovirus vaccine strains detected in stool specimens of immunodeficient children in South Africa Diagn Microbiol Infect Dis 2006 54 23 30 10.1016/j.diagmicrobio.2005.08.011 16290028

[B5] Pavlov DN Van Zyl WB Van Heerden J Kruger M Blignaut L Grabow WO Ehlers MM Prevalence of vaccine-derived polioviruses in stools of immunodeficient children in South Africa J Appl Microbiol 2006 101 1367 1379 10.1111/j.1365-2672.2006.03020.x 17105568

[B6] Fernandez-Ibieta M Ramos-Amador JT Aunon-Martin I HIV-infected children vaccination coverage and safety in a Western European cohort: a retrospective study Int J STD AIDS 2007 18 351 353 10.1258/095646207780749763 17524201

[B7] Tejiokem MC Gouandjika I Beniguel L Zanga MC Tene G Gody JC Njamkepo E Kfutwah A Penda I Bilong C Rousset D Pouillot R Tangy F Baril L HIV-infected children living in Central Africa have low persistence of antibodies to vaccines used in the Expanded Program on Immunization PLoS One 2007 2 e1260 10.1371/journal.pone.0001260 PMC2093997 18060056

[B8] PNUD Atlas de la République Centrafricaine sur les Indicateurs du VIH et du SIDA in Enquête à indicateurs multiples couplée avec la sérologie VIH et anémie en RCA, 2006 2008

[B9] Gouandjika-Vasilache I Akoua-Koffi C Begaud E Dosseh A No evidence of prolonged enterovirus excretion in HIV-seropositive patients Trop Med Int Health 2005 10 743 747 10.1111/j.1365-3156.2005.01454.x 16045460

[B10] Hennessey KA Lago H Diomande F Akoua-Koffi C Caceres VM Pallansch MA Kew OM Nolan M Zuber PL Poliovirus vaccine shedding among persons with HIV in Abidjan, Cote d'Ivoire J Infect Dis 2005 192 2124 2128 10.1086/498166 16288377

[B11] Giorgi JV Ianossy G, Autran B, Miedema F CD4 counts as surrogate makers for AIDS Immunodeficientcy in HIV infection and AIDS 1992 S. Karger, Switzerland 13

[B12] Menard D Mandeng MJ Tothy MB Kelembho EK Gresenguet G Talarmin A Immunohematological reference ranges for adults from the Central African Republic Clin Diagn Lab Immunol 2003 10 443 445 10.1128/CDLI.10.3.443-445.2003 PMC154963 12738646

[B13] WHO Manual for virological investigation of poliomyelitis 2004 Geneva, World Health Organisation

[B14] Wood DJ Hull B L20B cells simplify culture of polioviruses from clinical samples J Med Virol 1999 58 188 192 10.1002/(SICI)1096-9071(199906)58:2<188::AID-JMV15>3.0.CO;2-H 10335869

[B15] Guillot S Caro V Cuervo N Korotkova E Combiescu M Persu A Aubert-Combiescu A Delpeyroux F Crainic R Natural genetic exchanges between vaccine and wild poliovirus strains in humans J Virol 2000 74 8434 8443 10.1128/JVI.74.18.8434-8443.2000 10954543 PMC116354

[B16] Balanant J Guillot S Candrea A Delpeyroux F Crainic R The natural genomic variability of poliovirus analyzed by a restriction fragment length polymorphism assay Virol 1991 184 645 654 10.1016/0042-6822(91)90434-D 1679577

[B17] Thompson JD Higgins DG Gibson TJ CLUSTAL W: improving the sensitivity of progressive multiple sequence alignment through sequence weighting, position-specific gap penalties and weight matrix choice Nucleic Acids Res 1994 22 4673 4680 10.1093/nar/22.22.4673 PMC308517 7984417

[B18] Borkowsky W Rigaud M Krasinski K Moore T Lawrence R Pollack H Cell-mediated and humoral immune responses in children infected with human immunodeficiency virus during the first four years of life J Pediatr 1992 120 371 375 10.1016/S0022-3476(05)80899-6 1538282

[B19] Kroon FP van Dissel JT Labadie J van Loon AM van Furth R Antibody response to diphtheria, tetanus, and poliomyelitis vaccines in relation to the number of CD4+ T lymphocytes in adults infected with human immunodeficiency virus Clin Infect Dis 1995 21 1197 1203 10.1093/clinids/21.5.1197 8589143

[B20] Ryder RW Oxtoby MJ Mvula M Batter V Baende E Nsa W Davachi F Hassig S Onorato I Deforest A Kashamuka M Heyward WL Safety and immunogenicity of bacille Calmette-Guerin, diphtheria-tetanus-pertussis, and oral polio vaccines in newborn children in Zaire infected with human immunodeficiency virus type 1 J Pediatr 1993 122 697 702 10.1016/S0022-3476(06)80007-7 8496745

[B21] Moss WJ Clements CJ Halsey NA Immunization of children at risk of infection with human immunodeficiency virus Bull World Health Organ 2003 81 61 70 12640478 PMC2572316

[B22] Halsey NA Pinto J Espinosa-Rosales F da Silva E Khan AJ Webster AD Minor P Dunn G Asturias E Hussain H Pallansch MA Kew OM Winkelstein J Sutter R Search for poliovirus carriers among people with primary immune deficiency diseases in the United States, Mexico, Brazil, and the United Kingdom Bull World Health Organ 2004 82 3 8 15106294 PMC2585894

